# The Coronation and the Weather

**Published:** 1902-06-21

**Authors:** 


### The Hospital

A Journal of

The Medical Sciences and Hospital Administration.

Vol. XXXII.—No. 821. Edited by Sir Henry Burdett, K.C.B., and Solomon C. Smith, M.D., M.R.C.P. [June 21, 1902.

### THE CORONATION AND THE WEATHER.

The American diplomatist, whose professional desire to be agreeable enabled him to say no more—after a few weeks of sojourn in this country, and in reply to an inquiry as to his appreciation of the English climate—than that so far he had not seen any, he had seen nothing but weather, would this year have been able to plead an unusual degree of justification for his answer. We mostly have short memories about such matters, with the result that many people are now talking as if a wet and inclement June were an extraordinary phenomenon, whereas, in point of fact, it is one of which both our own and previous generations have had ample experience. Professor Laughton has appropriately published some extracts from the diary of a naval officer who served in Russell's fleet at La Hogue, and who described the midsummer day of 1692, a year in which, as Macaulay tells us, no fruit ripened in England, and in which the French army was well-nigh drowned in the trenches before Namur, as bearing more resemblance to midwinter. The Professor has also reminded us of the conditions of July, 1588, when Queen Elizabeth was able to commemorate the destruction of the Spanish Armada by a medal bearing the inscription *Deus afflavit et dissipati sunt.* The records of rainfall which have now for many years been preserved by the organisation established by the late Mr. Symons, and continued by Mr. Sowerby Wallis and Dr. Hill, are very little studied by the general public, but they extend over a period sufficiently long to include many wet summers.

There are yet some days to elapse before the great event of the Coronation, the event on which we have all fondly hoped that the skies would smile their brightest. Such hopes may yet be fulfilled; but it is our business, as prudent folk, to be prepared for disappointment. We are therefore face to face with a reasonable expectation that the hundreds of thousands of people who, on the 26th and 27th, will assemble in the streets of London, may be rained upon, and that an enormous proportion of them will be compelled to wait, perhaps for hours, in wet clothes and sodden foot gear, and without any possibility of keeping themselves warm by movement. The police regulations, as far as they are intelligible, appear to aim mainly at two results; first, at collecting the spectators together at an unreasonably early hour; and, secondly, at hindering and delaying their dispersal when the ceremony of the day is over. The advertised prices of seats to view the processions will be prohibitory to thousands of people, and, considering the expensive character of the structures involved, could hardly be reduced in any great degree. It follows that the immense majority of intending spectators will be fully exposed to the weather, whatever it may be, and even the protection afforded by umbrellas will scarcely be permitted by bystanders, whose necks would receive the streams running down from their ribs, a dire experience which was once familiar to those who rode upon the outsides of stage coaches. All the better class of seats will, it is said, be covered; but, judging from casual observation, the covering in many cases will be of a somewhat inefficient character, and its utility almost entirely dependent upon the direction of the wind. In these circumstances it becomes highly necessary to consider by what means the dangers incidental to exposure to cold and wet may be minimised; and the answer would be, chiefly by the avoidance of wet feet and of fasting. In these days of cheap waterproof cloaks and coats it will be comparatively easy to keep the general clothing at least so dry as not to cool the body dangerously by evaporation; and the chief point to be insisted upon should be waterproof foot coverings, such as are afforded by the things our trans-Atlantic cousins are accustomed to call "gums," but which are better known amongst ourselves by the fuller style of indiarubber goloshes. Next to dry feet, especially for children and young people, we should place a sufficient store of food, and an avoidance of the excessive bodily fatigue which would certainly be incidental to an effort to see too much, to be standing for hours waiting for the procession, and at night to be patrolling the streets for mile after mile for the purpose of seeing an endless series of illuminations, all of which will present a considerable family resemblance to all others. It is impossible to think without anxiety of the consequences which must follow from the prolonged exposure of thousands of people, many of whom, such as delicate women, adolescents of both sexes, and children, might fairly be described as "weakly," to a long day of excessive fatigue and of possible fasting, not to add of probable wet and cold; and we cannot too strongly urge upon those who intend to be spectators that they should use all available means of protecting themselves against the risks necessarily incidental to the undertaking. We presume that the general holiday will imply the closure of many of the places at which food is usually to be obtained; and perhaps the most important precaution will be to make provision against unduly prolonged fasting. The soldiers on duty are to be supplied with chocolate; and it would probably be difficult to suggest a better provision for those who come to see the show.

